# Effects of global warming on species with temperature‐dependent sex determination: Bridging the gap between empirical research and management

**DOI:** 10.1111/eva.13226

**Published:** 2021-04-04

**Authors:** Emma C. Lockley, Christophe Eizaguirre

**Affiliations:** ^1^ School of Biological and Chemical Sciences Queen Mary University London London UK

**Keywords:** climate change, reptiles, sea turtles, sex steroid hormones, temperature‐dependent sex determination

## Abstract

Global warming could threaten over 400 species with temperature‐dependent sex determination (TSD) worldwide, including all species of sea turtle. During embryonic development, rising temperatures might lead to the overproduction of one sex and, in turn, could bias populations’ sex ratios to an extent that threatens their persistence. If climate change predictions are correct, and biased sex ratios reduce population viability, species with TSD may go rapidly extinct unless adaptive mechanisms, whether behavioural, physiological or molecular, exist to buffer these temperature‐driven effects. Here, we summarize the discovery of the TSD phenomenon and its still elusive evolutionary significance. We then review the molecular pathways underpinning TSD in model species, along with the hormonal mechanisms that interact with temperatures to determine an individual's sex. To illustrate evolutionary mechanisms that can affect sex determination, we focus on sea turtle biology, discussing both the adaptive potential of this threatened TSD taxon, and the risks associated with conservation mismanagement.

## 1 INTRODUCTION

The current rate of species loss is often referred to as the sixth mass extinction event in geological history (Barnosky et al., 2011). As climate change progresses and global temperatures continue to rise rapidly, understanding how species interact with their environment has become extremely important (Hoffmann & Sgrò, 2011; Neukom et al., 2019; Stocker et al., 2013; Visser, 2008). This is particularly true for over 400 fish and reptile species that have temperature‐dependent sex determination systems (TSD). For these species, incubation temperatures differentially trigger the pathways that lead to male and female gonad development (Charnov & Bull, 1977; Deeming et al., 1988). As global warming continues, the TSD mechanism could lead to heavily skewed offspring sex ratios towards one sex, which in turn threatens populations’ persistence (Eberhart‐Phillips et al., 2017; Laloë et al., 2014; Mitchell & Janzen, 2010). Whether TSD species will be able to withstand the rates of predicted temperature change and maintain viable sex ratios will depend on their adaptive potential (Eizaguirre & Baltazar‐Soares, 2014). Adaptive potential is defined as “the ability of populations/species to respond to selection by means of phenotypic or molecular changes” and interacts with population structure and demography (Eizaguirre & Baltazar‐Soares, 2014; Rey et al., 2020). Even though knowledge on the adaptive potential of TSD is essential for conservation management, it remains uncharacterized in most species, such as sea turtles (Santidrián Tomillo & Spotila, 2020).

To understand pressures on sea turtles, it is essential to acknowledge that in addition to anthropogenic climate change, they face cumulative impacts from other human‐induced stressors (Hawkes, Broderick, Coyne, et al., 2007; Hawkes, Broderick, Godfrey, et al., 2007; McMahon & Hays, 2006; Witt et al., 2010), such as coastal development (Kaska et al., 2013; Von Holle et al., 2019), fisheries bycatch (Fossette et al., 2014; Senko et al., 2014) and illegal harvest of both eggs and adults (Senko et al., 2014; Tomillo et al., 2008). As a consequence, many populations are already depleted or in decline, and subject to extensive conservation management plans (Hamann et al., 2010; Mortimer & Donnelly, 2008; Wallace et al., 2013). The actions to protect sea turtles from the effects of global warming are further limited by the inability to determine a neonate's sex nonlethally and the difficulty in justifying the sacrifice of individuals from endangered populations, both of which restrict TSD research in this taxon. As such, most interest in trying to quantify how sea turtles will adapt to climate change has focused on nesting behaviour such as phenological changes and site selection (Mazaris et al., 2013; Patrício et al., 2017; Refsnider, Bodensteiner, et al., 2013; Refsnider, Warner, et al., 2013; Reneker & Kamel, 2016). Similarly, most approaches to mitigate the effects of rising temperatures have involved human manipulation of nest temperatures through, for example, relocation (either in situ or in hatcheries) and shading (DeGregorio & Williard, 2011; Mrosovsky, 2006; Tuttle & Rostal, 2010). Here, we review the current knowledge and highlight the importance of the adaptive potential of TSD mechanisms, by bridging empirical research gained from TSD model species with the more practical management of wild populations of sea turtles. We chose to highlight molecular and physiological responses, which we consider to be under‐represented in sea turtle research in comparison with behavioural adjustments (Patrício et al., 2017; Reneker & Kamel, 2016). Finally, we discuss how failing to consider the adaptive potential and underlying mechanisms of TSD in sea turtles could lead to inappropriate management decisions.

## 2 TSD PATTERNS AND ENVIRONMENTAL COVARIATES

TSD species have no sex chromosomes. Instead, sex‐determining genes are scattered across the genomes, and male‐ or female‐determining pathways are triggered by temperature during a thermosensitive period of development (Bachtrog et al., 2014; Charnov & Bull, 1977; Shen & Wang, 2014). This mode of sexual development was first reported in the common agama lizard, *Agama agama* in 1966 (Charnier, 1966). It has since been confirmed as the sex‐determining mechanism of several reptile lineages, including the tuatara, crocodilians and turtles (Cree et al., 1995; Janzen & Paukstis, 1991).

**FIGURE 1:**
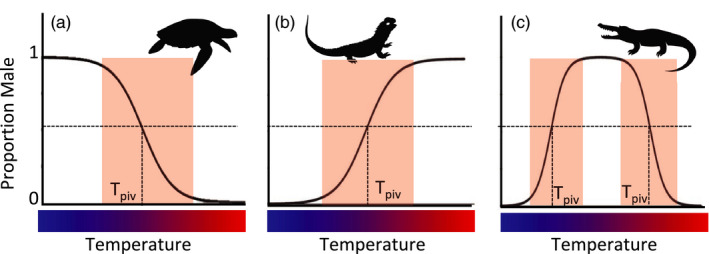
The three patterns of temperature‐dependent sex determination: (a) type Ia, as seen in sea turtles; (b) type Ib, as known in tuatara; and (c) type II, as present in crocodilians. The pivotal temperature (*T*_piv_) is the temperature at which an even proportion of males and females is produced. Red denotes the transitional range of temperatures, where both sexes can be produced, generally defined as between 5% and 95% of one sex

Different patterns of TSD exist (Figure 1). In type Ia TSD, seen in most turtle species, males develop at cooler temperatures while females are produced under warmer conditions (e.g. the painted turtle, *Chrysemys picta*, Bull & Vogt, 1979). In type Ib, this pattern is reversed, and males are produced at warm temperatures (e.g. the tuatara, *Sphenodon punctatus*, Cree et al., 1995). Finally, species with type II TSD, common to all crocodilians, produce males at intermediate temperatures and females at both hot and cold extremes (e.g. the American alligator, *Alligator mississippiensis*, Ferguson & Joanen, 1983; González et al., 2019). Under constant incubation temperatures, the TSD thermal response curve is described by i) a pivotal temperature, at which an equal number of embryos within a clutch develop as males and females, and ii) the range of temperatures under which either male or female offspring may be produced, known as the transitional range of temperatures (Figure 1; Girondot, 1999; Mrosovsky & Pieau, 1991).

Genetic sex determination (GSD) and TSD are often considered to be mutually exclusive mechanisms, but instead should be regarded as two ends of a continuum, with environmental variation interacting with genetic mechanisms to different extents across species (Bachtrog et al., 2014; Holleley et al., 2015, 2016; Pen et al., 2010; Quinn et al., 2007, 2011). For instance, the montane lizard, *Bassiana duperreyi*, has heteromorphic sex chromosomes, and eggs incubated at warm temperatures, characteristic of lowland environments, produce equal numbers of male and female offspring. Yet, when eggs are incubated at cool temperatures representative of high altitude summers, the ratio is skewed towards 70% male hatchlings, suggesting that temperature can partly override genetic triggers in this species (Shine et al., 2002). In mammals, which have GSD, maternal condition can alter sex ratios (Grant, 2007; Sheldon & West, 2004; Trivers & Willard, 1973), moderated by the environmental conditions during the breeding and gestation periods (Edwards et al., 2019).

Temperature is the primary determinant of gonad differentiation in TSD species, and the thermal environment of nests varies with substrate albedo (Hays et al., 2001), shading (Refsnider, Bodensteiner, et al., 2013; Refsnider, Warner, et al., 2013) and nest depth (Telemeco et al., 2009; Santidrián Tomillo et al., 2017), which can all influence egg development. These variables differ depending on individual nest site selection and seasonality and thus introduce variation into offspring sex ratios within and among populations (Reneker & Kamel, 2016). It is also known that precipitation and humidity interact with thermal conditions and hence influence TSD. For instance, the relationship between rainfall and sex ratios has generally been attributed to the cooling effect of rain on the temperature of nesting substrate thanks to evaporative cooling (Godfrey et al., 1996; Houghton et al., 2007; Lolavar & Wyneken, 2015; Matsuzawa et al., 2002). An argument has recently also been made to suggest that humidity itself has an effect on sex ratios beyond that of temperature alone (Lolavar & Wyneken, 2017, 2020). While these studies have not yet robustly demonstrated the effect of humidity on TSD, they do illustrate several points. Firstly, temperature does not act in isolation in natural environments. Secondly, we still do not fully understand all the factors that influence sex determination in TSD species. As such, we should consider the potential for elements of the thermal response curve (e.g. the pivotal temperature and transitional range of temperatures) to vary from traditional values—either from plastic responses or as a result of adaptive evolution (Santidrián Tomillo & Spotila, 2020).

## 3 THE EVOLUTIONARY SIGNIFICANCE OF TSD

A comprehensive theory explaining the selective advantage of TSD still seems to evade researchers (Pen et al., 2010; Quinn et al., 2011; Sarre et al., 2004). While the random segregation of sex chromosomes in species with GSD reflects parents’ equal chromosomal investment in male and female offspring, conforming to frequency‐dependent selection, no such theory exists to easily explain the evolutionary significance of TSD (Fisher, 1930). Instead, three relatively robust hypotheses for the evolution of TSD have been suggested, with varying levels of support; (i) the Charnov–Bull model of differential fitness, (ii) the “Mighty Males” hypothesis, and (iii) the phylogenetic inertia hypothesis (Charnov & Bull, 1977; Girondot & Pieau, 1999; Janzen & Phillips, 2006; Rollinson, 2019; Shine, 1999).

The Charnov–Bull model of differential fitness suggests that sex‐specific advantages are associated with particular thermal environments and that the TSD mechanism ensures the production of sexes at their optimal temperatures (Charnov & Bull, 1977). The theory requires a heterogeneous environment, in time or space, where males and females benefit from different conditions (Charnov & Bull, 1977). Sex‐specific benefits from particular thermal environments emerge through different mechanisms. For instance, incubation temperature may (i) affect fitness proxies, such as growth rate or size, (ii) correlate with future conditions that offspring will experience, or (iii) affect developmental rates and timing of emergence (Janzen & Phillips, 2006; Shine, 1999). Recently, it was suggested that sex‐specific fitness may stem from bimodal age at maturity distributions, as TSD species show greater dimorphism in this trait than GSD species (Bókony et al., 2019).

In TSD species, the effects of temperature and sex overlap, and therefore, it is challenging to disentangle their relative contributions to an individual's fitness (Rhen & Lang, 2004). This problem has generally been overcome with the use of exogenous hormone manipulation experiments, whereby a given sex is artificially forced to develop at temperatures that would not otherwise enable its production. One of the best examples of such an experiment focused on the jacky dragon, *Amphibolurus muricatus* (Warner & Shine, 2008). Eggs from this agamid lizard were incubated at a range of temperatures, and half of them were treated with an aromatase inhibitor that forced embryos to develop as male, regardless of their thermal environment. This manipulation decoupled the effects of sex and temperature on fitness and revealed that lifetime reproductive success was greater for males that were incubated at natural male‐producing temperatures (Warner & Shine, 2008). Importantly, hormone treatment had no effect on the morphology or survivorship of juvenile jacky dragons, indicating no effect of the treatment itself. Since this seminal study, further experiments have produced similar results across other agamid species (Steele & Warner, 2020). While this reproducibility gives weight to the Charnov–Bull hypothesis, whether these results can be extrapolated to all TSD species, and particularly long‐lived ones, remains unclear (Steele & Warner, 2020). This is because there are conflicting results among experiments testing the Charnov–Bull model (Janzen & Phillips, 2006). For instance, studies using the diamondback terrapin *Malaclemys terrapin* failed to find support for the Charnov–Bull model (Morjan & Janzen, 2003). As such, caution is needed when presenting the Charnov–Bull model as a universal explanation for TSD in reptiles (Janzen & Phillips, 2006).

Recently, Rollinson (2019) proposed an alternative theory for the evolution of TSD, referred to as the “Mighty Males” hypothesis, based on the maternal condition hypothesis described by Trivers and Willard (1973). The original hypothesis posits that females’ lifetime reproductive success is mostly constrained by the number of gametes they produce and, as such, even lower quality female offspring produced under suboptimal conditions should not suffer a reduction in lifetime reproductive fitness. On the other hand, males’ reproductive success is limited by their ability to compete for mating opportunities, and therefore, male offspring should be produced under the environmental conditions that maximize their overall fitness (Trivers & Willard, 1973). Given these constraints are related to sexual reproduction, independently of TSD, and given that temperature affects a wide range of traits such as size, developmental rate or embryonic mortality, Rollinson (2019) proposes that males should be produced at the temperatures that maximize the fitness potential of these traits, while females should be produced at the suboptimal extremes. As mortality increases with high temperatures, the Mighty Males hypothesis can be easily applied to type II (female–male–female) and type Ia (male–female) TSD species (Santidrián Tomillo & Spotila, 2020). On the other hand, it may not apply to type Ib (female–male) TSD species, unless evolutionary mechanisms exist that can significantly push back the upper temperature limits for mortality. Type Ib is rare in nature, existing in the tuatara and shorter‐lived squamata groups, and may require an alternative explanation.

Finally, the difficulty in determining the evolutionary significance of TSD may stem from phylogenetic inertia and the possibility its adaptive significance may no longer be detectable 300 million years after it evolved (Janzen & Krenz, 2004; Janzen & Phillips, 2006). After phylogenetically reconstructing the evolution of sex determination in over 400 species of squamata, many examples of transitions from TSD to GSD were identified, but there were no cases where this direction was reversed (Pokorná & Kratochvil, 2009). It was therefore suggested that sex chromosomes may evolve when sex‐specific genes are coupled with genes that provide a selective advantage to that sex. As the association between these genes strengthens and rates of recombination decrease, the conditions are met for sex chromosomes to evolve (Bachtrog et al., 2014; Muralidhar & Veller, 2018).

## 4 HERITABILITY, DEVELOPMENT AND MOLECULAR PATHWAYS OF SEX DETERMINATION

Despite very different sex determination mechanisms across vertebrate taxa, similar genetic pathways underpin the molecular foundations of gonad differentiation. This is consistent with conserved patterns of gonad development across vertebrates, with gonads being initiated as a bipotential genital ridge in both GSD and TSD species. From a developmental perspective, the genital ridge is formed from the coelomic epithelium, underlying mesenchymal cells and germ cells, which migrate into the ridge from the embryonic yolk sac (Morrish & Sinclair, 2002). As the somatic and germ cells proliferate, the genital ridge expands until the cell fate commits to developing as testes or ovaries. The differentiation of testes necessitates the development of the primary sex cords into testis cords, whereas the formation of ovaries requires the thickening of the coelomic epithelium while the primary sex cords disappear (Morrish & Sinclair, 2002). Importantly, depending on the species and the TSD pattern, the molecular mechanisms of the sex‐determining period can either end before signs of gonad differentiation appear, or may overlap with the early stages of gonad differentiation (Morrish & Sinclair, 2002).

Overall, there is no doubt about the role of temperature in sex determination of TSD species, yet individual responses, reflected in traits such as the feminization threshold of embryos, are underpinned by a genetic contribution with some levels of heritability, estimated in the painted turtle, *Chrysemys picta*, to be as high as *h* ^2 ^= 0.35 (McGaugh et al., 2011). It is therefore not surprising that studies have focused on elucidating the molecular mechanisms of sex determination (Martínez‐Juárez & Moreno‐Mendoza, 2019). Conventionally, genes are classified based on their GSD sex‐specific pathways and probably all known mammalian sex‐determining genes have now been tested for their role in TSD (Rhen & Schroeder, 2010). A very thorough gene‐based overview can be found in Martínez‐Juárez and Moreno‐Mendoza (2019). In brief, the expression of genes such as *Foxl2* (Forkhead box protein L2) and *Rspo1* (encoding the protein R‐spondin‐1) becomes greater in the female‐determining pathway, but these are also detectable in the male cascade during early development (Elf, 2003). Furthermore, *Dax1* (dosage‐sensitive sex reversal, adrenal hypoplasia critical region), *Sf1* (steroidogenic factor 1) and *Wnt4* (wingless‐type MMTV integration site family, member 4), all involved in female development and the repression of male traits, also appear to contribute to the sex determination of TSD species (Shoemaker & Crews, 2009). Conversely, in the male pathway, genes necessary for testicular differentiation include *Sox9* (SRY‐box transcription factor 9), the anti‐Mullerian hormone (*AMH*) and *Dmrt1* (doublesex and Mab‐3‐related transcription factor 1). The expression of *Sox9*, a gene that belongs to the same HMG‐box transcription factor as *Sry* (sex‐determining region Y), is detectable in early gonad development and becomes restricted to the developing testis at the end of the thermosensitive period (reviewed in Rhen & Schroeder, 2010). Noteworthy, the expression of *Sry* remains elusive in reptiles. *Dmrt1* is particularly interesting and shows temperature‐dependent sex‐specific expression that precedes gonadal sex differentiation. *Dmrt1* expression changes with shifts in temperature in a sex‐specific manner and also responds to the presence of aromatase inhibitors—regulation of this gene has been shown to be necessary in order to initiate male development in *Trachemys scripta* (Ge et al., 2017).

While the identification of these genes follows a candidate gene approach, in more recent years studies of TSD have focused on gene discovery. Along this vein, several new candidates have emerged. A single nucleotide polymorphism in *Cirbp* (cold‐inducible RNA‐binding protein) was associated with transcript levels in the embryonic gonads of the snapping turtle (*Chelydra serpentina*) during specification of gonad fate and hatchling sex (Rhen & Schroeder, 2017; Schroeder et al., 2016). The A allele was induced in embryos exposed to a female‐producing temperature, while expression of the C allele did not differ between female‐ and male‐producing temperatures. As such, AA homozygotes were more likely to develop ovaries than the CC homozygotes which all developed as males, with the AC heterozygotes standing at an intermediate frequency. From an ecological perspective, it is worth noting that changes in allele frequencies in *Cirbp* were detected at small and large geographical scales, suggesting local adaptation. Such patterns of local adaptation would be expected to result in higher pivotal temperatures in warmer regions, possibly mediated by resistance thresholds to the development of the high‐temperature sex, linked to genetic underpinnings. On the other hand, in the central bearded dragon (*Pogona vitticeps*), a species in which chromosomal sex determination is overridden at high temperatures, sex‐reversed females are produced when an intron is retained in the mature transcripts from each of two Jumonji family genes, *Jarid2* (Jumonji and AT‐rich interaction domain‐containing 2) and *Jmjd3* (Jumonji domain‐containing 3, histone lysine demethylase). This intron retention was observed only in females that have been sex‐reversed by temperature, not in classic chromosomal females or males (Deveson et al., 2017). Similarly to *Cirbp* in *C. serpentina*, if the central bearded dragon has evolved mechanisms to regulate the overproduction of a given sex, we should be able to observe spatial and temporal variation of this intron loss in relation to temperature clines.

De novo genome sequencing has also introduced elements of gene and mechanism discovery. This has been the case with the assembly of American alligator genomes, which, combined with RNA sequencing and models of CTCF‐mediated chromatin looping, identified genomic regions that were significantly enriched for genes with female‐biased expression in developing gonads after the thermosensitive period (Rice et al., 2017). This approach demonstrated that oestrogen signalling is a major driver of female‐biased gene expression and holds promises for comparative genomics of TSD species.

Finally, a common pattern that has emerged from genomic studies is that the discovery of new TSD‐associated genes does not appear to be independent of epigenetic mechanisms. For instance, the DNA methylation dynamics of the *Dmrt1* promoter region are tightly correlated with temperature and could mediate the impact of temperature on sex determination (Ge et al., 2018). In *T. scripta*, Ge et al. (2018) demonstrated how the epigenetic regulator *Kdm6b* demethylates the histone H3 lysine 27 (*H3K27*) at the *Dmrt1* promoter region, in a process that results in male sex determination. It appeared though that *Kdm6b* is not in itself responsive to temperature, and as such, the fundamental thermal trigger of this pathway remains unknown. Similarly, *Jarid2* is a component of the master chromatin modifier polycomb repressive complex 2, and the mammalian sex‐determining factor *Sry* is directly regulated by an independent but closely related Jumonji family member (Deveson et al., 2017). The authors proposed that the alteration of *Jarid2*/*Jmjd3* function by intron retention alters the epigenetic landscape to override chromosomal sex‐determining cues, triggering sex reversal at extreme temperatures (Deveson et al., 2017).

It is difficult to predict the nature of selection exerted by global warming, as directional selection would be expected under a constant increase in temperature, but a predicted increased frequency of extreme events should result in fluctuating selection. When selection is constrained, then phenotypic plasticity may allow species to respond to their environment (Chevin et al., 2010). Indeed, while epigenetics, as mechanisms of phenotypic plasticity, may appear distant to practical conservation, their relevance has recently been framed in the light of management decisions (Rey et al., 2020). Altogether, understanding the mechanisms underlying phenotypic plasticity is a valuable direction of research in conservation management. In particular, here we suggest that increased consideration of endocrinology and sex steroid hormones would benefit the conservation of TSD species.

## 5 PLASTICITY AND SEX STEROID HORMONES IN TSD SPECIES

Physiological plasticity includes a series of mechanisms by which organisms can match their phenotypes to their environmental conditions, for instance via the endocrine system and hormone regulation (Chevin et al., 2010; Gienapp et al., 2008; Merilä & Hendry, 2014). In the specific context of phenotype–environment matching, evolutionary theory suggests that the populations demonstrating the greatest levels of plasticity in beneficial traits might have the highest potential of persisting in the face of climate change (Meyers & Bull, 2002). This is because if the cost of plasticity is low, it will reduce the range of conditions under which extinction is inevitable (Chevin et al., 2010; Sanford & Kelly, 2011). Plasticity could thus maintain populations until adaptive evolution emerges and improves the phenotype–environment match (Lande, 2009; Reusch, 2014).

Sex steroid hormones, such as oestrogens and their precursors, androgens, interact with the molecular pathways that control gonad differentiation and are fundamental for regulating gonad development across a wide variety of taxa (spanning amphibian (Ko et al., 2008), bird (Nakabayashi et al., 1998), fish (Wang et al., 2007), mammals (Uhlenhaut et al., 2009) and reptiles (Barske & Capel, 2010)). Oestrogens repress *Sox9* expression, preventing the differentiation of male gonad‐specific Sertoli cells in both GSD (mouse, Uhlenhaut et al., 2009) and TSD species (*T. scripta*, Barske & Capel, 2010). Conversely, the development of the müllerian ducts, which provide the structure for female gonads, correlates with changes in oestrogens in mammals, birds and reptiles (Dodd & Wibbels, 2008). In TSD species, the oestrogen 17β‐oestradiol replicates the effect of temperature in instigating demethylation of histone H3 lysine 27 (*H3K27*) at the *Dmrt1* promoter region, resulting in female gonad development (Ge et al., 2018). Moderating embryonic exposure to sex steroid hormones is thus likely to be an effective mechanism for conferring plasticity to the TSD thermal response curve (Bowden et al., 2000; Carter et al., 2017).

The influence of androgens and oestrogens on embryonic sex independently of temperature in TSD species has been extensively reviewed (Bowden & Paitz, 2018; Elf, 2003). To date, research exploring the relationship between steroid hormones and TSD pathways has largely focused on oestradiol, testosterone and the enzyme aromatase (Box 1). Studies began with in vitro manipulation experiments which showed that exogenous application of oestradiol produced female offspring at male‐producing temperatures (Crews et al., 1989, 1991; Wibbels et al., 1991). At the same time, the application of aromatase inhibitors such as fadrozole, which prevent the synthesis of oestradiol, has repeatedly resulted in male offspring (Rhen & Lang, 1995; Warner et al., 2017; Wibbels & Crews, 1994). Patterns of gonad development in response to exogenous hormone application have not, however, always been predictable and negative results have been reported, along with incidences where exogenous application of oestradiol unexpectedly produced male offspring (Janes et al., 2007; Warner et al., 2014).

BOX 1 **An overview of Aromatase**

Aromatase is an enzyme encoded by the *Cyp19a1* gene (Strauss & FitzGerald, 2018), which is part of the cytochrome P450 superfamily. This protein is the only known enzyme to catalyse the conversion of androgens to oestrogens, a process that occurs throughout all vertebrate taxa. The P450 superfamily is an ancient lineage of genes that diverged early in the evolution of vertebrates (Boon & Simpson, 2012; Nelson et al., 2013; Simpson, 2004), with gonadal synthesis of oestrogens originating 500 mya (Lange et al., 2002). *Cyp19a1* is highly conserved (Conley & Hinshelwood, 2001). In fish and reptiles, there are two aromatase isomorphs encoded by the *Cyp19a1* and *Cyp19b1* genes and expressed in the gonads and brain, respectively (Boon & Simpson, 2012). For most species, the majority of oestrogen biosynthesis occurs in the gonads, with biosynthesis in the brain associated with behaviour (Simpson, 2004). In comparison, a single gene encodes human aromatase, with tissue‐specific promoter regions found on exon 1 of this gene, enabling its biosynthesis to occur in a greater range of tissues (Bulun et al., 2004; Sebastian & Bulun, 2001). The upregulation of *Cyp19a1* in the gonads is required for ovarian differentiation in fish (Guiguen et al., 2010), birds (Smith et al., 1997) and reptiles (Jeyasuria & Place, 1998), but not in mammals, where the knockout of *Cyp19a1* does not prevent ovaries from developing (Fisher et al., 1998).

The apparent inconsistencies that are reported both between in vitro experiments and across species can be partially explained by interactions between exogenous hormones and temperature. Oestradiol and temperature produced more female embryos than would be expected by each treatment alone near the pivotal temperature of the red‐eared slider turtle *T. scripta* (Wibbels et al., 1991). Under extreme natural incubation temperatures (>2**°**C above the 75‐year nesting site average), exogenous application of oestradiol and fadrozole had no effect on painted turtle sex ratios (Warner et al., 2017). Yet, under average seasonal conditions exogenous oestradiol produced more female offspring, and fadrozole more male offspring, than controls (Warner et al., 2017). These results suggest that temperature and hormones may have a dose‐dependent and interactive effect on sex determination pathways (Wibbels et al., 1991).

**FIGURE 2:**
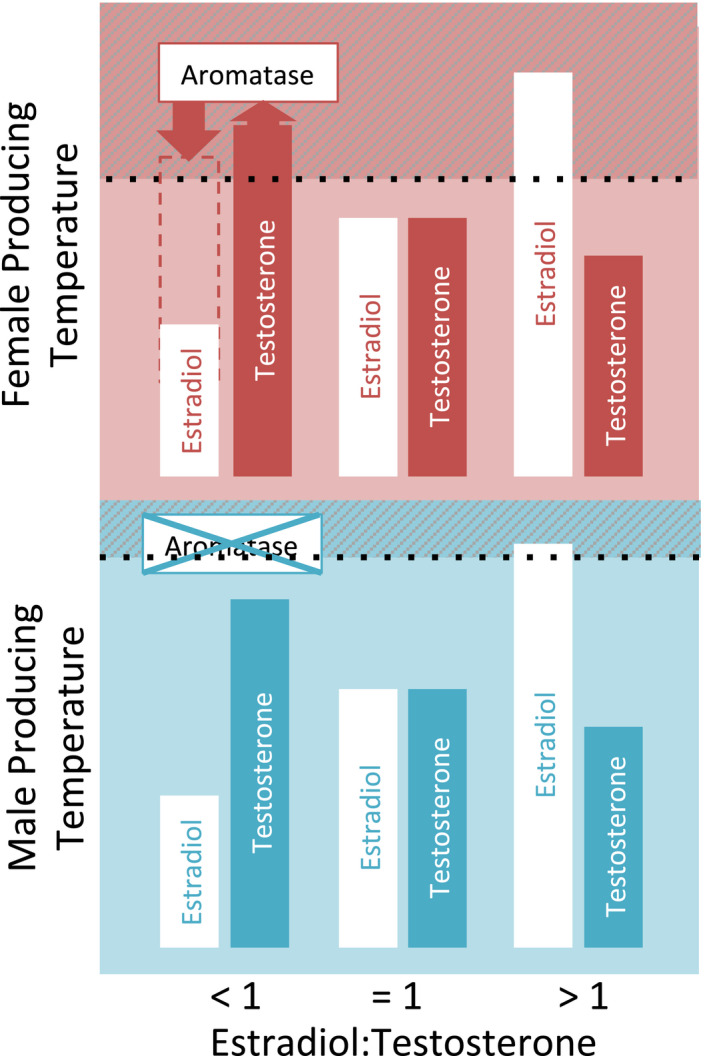
TSD sex‐determining pathways are triggered by an interaction between temperature and maternally derived sex steroid hormones, which must achieve a threshold for female gonad development (shaded area/dashed line). Under this hypothesis, at female‐producing temperatures, temperature acts on thermally sensitive pathways, and the threshold of oestradiol concentration required for female gonad development is reduced. In loggerhead sea turtles, the ratio of oestradiol: testosterone of yolk primed by maternal transfer can be skewed in favour of either hormone (Lockley et al., 2020). Thus, at female‐producing temperatures, the feminization threshold can be achieved either directly from maternally derived oestradiol (when E:T > 1) or from the biosynthesis of testosterone by aromatase, which is expressed at female‐producing temperature (when E:T < 1). When the oestradiol:testosterone ratio is equal to one, product feedback inhibition of aromatase activity can prevent the biosynthesis of testosterone. In this case, the feminization threshold is not achieved, and male offspring are produced. At male‐producing temperatures, the feminization threshold is higher due to the lack of pressure on thermal triggers, aromatase is not expressed, testosterone is not synthesized into oestradiol, and thus, feminization requires high concentrations of maternally derived estradiol

In oviparous species, a principal conduit of hormone transfer from mother to offspring is the egg yolk (Radder, 2007; Schwabl, 1993). It provides material with which to prime the reactions associated with the molecular cascades that trigger gonad differentiation. In European sea bass *Dicentrarchus labrax*, exposure to male‐producing temperatures results in methylation of the *Cyp19a1* gene and lower aromatase expression (Navarro‐Martín et al., 2011). Similar methylation patterns are also seen in red‐eared slider turtles *T. scripta* (Matsumoto et al., 2016). High levels of aromatase expression will increase the biosynthesis of oestradiol from testosterone, if this substrate is available and the reaction is not inhibited (Boon & Simpson, 2012). Interestingly, the oestradiol: testosterone ratio within egg yolks at oviposition (i.e. before synthesis has occurred) varies across reptile species, having been recorded as both above and below 1, but rarely spanning an equal ratio (Radder, 2007), suggesting species‐specific regulation. Depending on species, endogenous oestradiol in yolks could act on female‐producing pathways directly, or indirectly through the aromatase synthesis of endogenous testosterone, to produce female hatchlings (Figure 2). However, in loggerhead sea turtles, the ratio of oestradiol: testosterone in egg yolks can favour either hormone, or be equal (Lockley et al., 2020). In a natural experiment where temperatures were controlled among nests, equal, low concentration, oestradiol: testosterone ratios produced male offspring, which the authors theorize is due to product‐feedback inhibition of aromatase (Figure 2, Lockley et al., 2020). Such reactions will be occurring within the yolk of eggs used for exogenous application studies. The interference with natural oestradiol: testosterone ratios that could occur in these experiments might thus explain those occasions where exogenous oestradiol application has produced male offspring (Janes et al., 2007; Warner et al., 2014).

A fundamental constraint of our knowledge generated by exogenous application studies is therefore that the biological relevance of results within natural systems is extremely limited (Bowden & Paitz, 2018). Field studies are invaluable for elucidating how this mechanism interacts with environment. When clutches of painted turtles were incubated at constant temperatures of 28°C, the endogenous oestradiol: testosterone ratio within egg yolks correlated with a seasonal shift in sex ratio from 72% male to 76% female (Bowden et al., 2002). This relationship resulted in thermal reaction curves systematically changing across a nesting season, in a manner that allowed sex‐specific phenotypic matching, enhancing the production of female offspring under warm conditions (Carter et al., 2017). Such a mechanism fits directly with the Charnov–Bull differential fitness hypothesis, allowing nesting females to maximize female offspring production under temperatures that could be most beneficial to them. In addition, it is consistent with the “Mighty Males” hypothesis, favouring the production of female offspring under warm conditions that may increase mortality rates. This mechanism, however, could accelerate the rate of female production as temperatures rise and may therefore represent an example of evolutionary suicide (Bowden et al., 2000).

## 6 TEMPERATURE‐DEPENDENT SEX DETERMINATION AND SEA TURTLES

**FIGURE 3:**
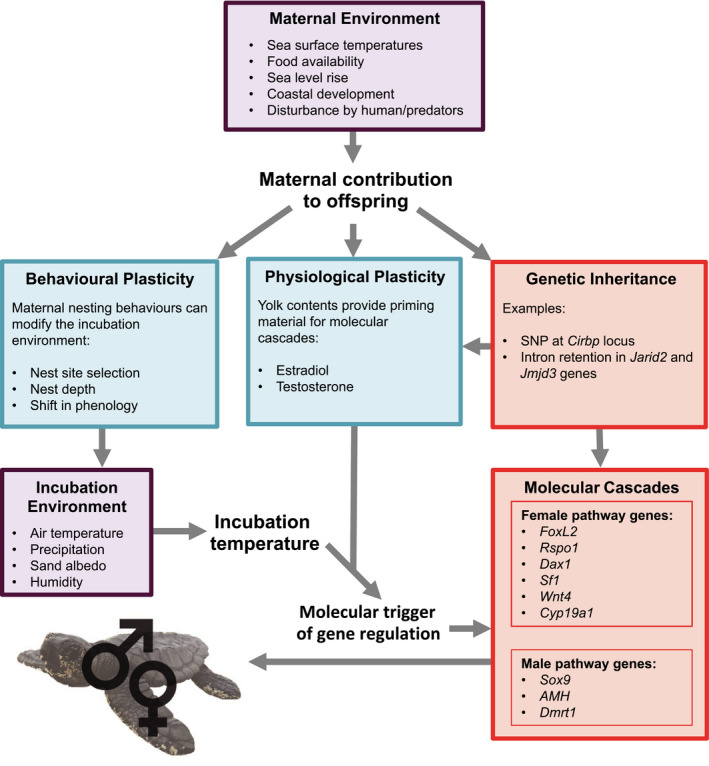
Integrative perspective on how environment and adaptive potential interact with molecular cascades to establish sex in sea turtles, a species with temperature‐dependent sex determination. Environmental cues within the maternal environment may trigger plastic responses (blue). Genetic inheritance (red) of specific genetic elements/alleles can lead to greater resistance to high temperatures. Together, behavioural and physiological plasticity influence the environment of the developing embryo, differentially triggering molecular cascades that result in male or female offspring

We have considered the many molecular pathways that contribute to the formation of gonads in TSD species, and outlined how sex steroid hormones can interact with temperature (Figure 3). We now focus on applying this gained knowledge to sea turtles and their management, in the light of global warming. Sea turtles are type Ia TSD species (Yntema & Mrosovsky, 1982), and thus, thermal projections across the coming century suggest that offspring sex ratios will become increasingly feminized (Hawkes, Broderick, Coyne, et al., 2007; Hawkes, Broderick, Godfrey, et al., 2007; Hawkes et al., 2009; Laloë et al., 2014; Tanner et al., 2019; Witt et al., 2010; Yntema & Mrosovsky, 1982). These projections, however, do not generally account for the adaptive potential of populations (Santidrián Tomillo & Spotila, 2020).

Sea turtles demonstrate a high degree of natal philopatry to their nesting sites, often with high female fidelity and male‐mediated gene flow among mating sites (Bowen & Karl, 2007; Lee et al., 2007; Levasseur et al., 2020; Meylan et al., 1990; Stiebens et al., 2013). This strong female philopatry creates genetic structure within a rookery that is prone to the evolution of local adaptation (Baltazar‐Soares et al., 2020; Stiebens et al., 2013). Female turtles return to nest at intervals ranging from 1 to 4 years, while males return more frequently and can even remain resident to the mating grounds (Arendt et al., 2012; Hays et al., 2010; Schofield et al., 2010). Different remigration intervals between males and females result in different operational sex ratios (the ratio of sexually active males to females at the nesting site a given time) than that of adult sex ratios, which are likely to be less female‐biased than offspring sex ratios (Hays et al., 2014). While the operational sex ratio and even mating strategies (e.g. Lee & Hays, 2004) may alleviate offspring sex ratio bias, a reduction in the total number of males can have implications for populations: it can lead to reduced genetic diversity (Frankham, 2005), increase potential for inbreeding and genetic drift (Hedrick & Kalinowski, 2000) and ultimately reduce a population's fitness and its adaptive potential (Reed & Frankham, 2003).

## 7 TSD IN SEA TURTLES: THE BARRIERS

Little is known about TSD in sea turtles as, historically, sexing individuals has required sacrifice and histological examination of gonads (e.g. Fuentes et al., 2017; Hamann et al., 2010; Wyneken et al., 2007). As most sea turtle populations are listed on the IUCN red list, the sacrifice of hatchlings is often restricted by local authorities. Over the years, several protocols have been developed to overcome this challenge, such as quantifying the oestradiol: testosterone ratio in hatchling plasma (green sea turtle: 96.7% overall accuracy, *n* = 30, Xia et al., 2011, loggerhead sea turtle: 94% accuracy for males and 100% accuracy for females, *n* = 28, Gross et al., 1995). Along the same approach, the expression of Anti‐Müllerian Hormone (*AMH*) seems to also effectively identify the sex of loggerhead sea turtle hatchlings, being expressed only in males (*n* = 59, Tezak et al., 2020). These protocols will now need to be upscaled to reach a high throughput, to lead a new era of research in the study of TSD and management of sea turtles.

Until large‐scale sexing techniques are possible, our overall knowledge of TSD thermal response curves in sea turtles is limited to laboratory incubation studies with small sample sizes (Godfrey et al., 1999; Mrosovsky, 1988; Mrosovsky et al., 1992; Rimblot et al., 1985). These studies mostly identified the pivotal temperature of sea turtle populations to lie around 29°C (but see McCoy et al., 1983; Wibbels et al., 1998). In more recent years, sophisticated modelling approaches have been used to refine the characteristics of thermal response curves (Abreu‐Grobois et al., 2020), and have also quantified variation among populations (Bentley et al., 2020). However, prior to these advances, 29°C was often used as an approximate pivotal temperature to estimate sex ratios of sea turtle populations in cases where sacrificing hatchlings was not possible, using mean nest temperature during the middle third of incubation as a proxy (see Table 1 for illustrative cases). Alternatively, studies have used the thermal response curves described specifically for the population of interest or a nearby neighbour, if these values have been reported (Table 1).

**TABLE 1:** Examples of studies that use thermal proxies to indirectly estimate offspring sex ratios, and the origin of the pivotal temperature (*T_p_*_iv_)­ used to estimate this

Species	Location	*T*_piv_ (°C)	Source of *T*_piv_
Green (Esteban et al., 2016)	Chagos Archipelago	29	Review (Ackerman, 1997)
Green (Broderick et al., 2001)	Ascension Island	29	Review (Ackerman, 1997)
Green (Booth & Freeman, 2006)	Heron Island, Australia	27.5	*T*_piv_ previously calculated for population (Booth & Astill, 2001)
Hawksbill (Esteban et al., 2016)	Chagos Archipelago	29	Review (Ackerman, 1997)
Hawksbill (Glen & Mrosovsky, 2004)	Antigua	29.2	Review (Mrosovsky & Pieau, 1991)
Leatherback (Santidrián Tomillo et al., 2014)	Costa Rica	29.4	*T*_piv_ previously calculated for same population (Binckley et al., 1998)
Loggerhead (Laloë et al., 2014)	Cabo Verde	29, 28.8 and 29.2	Mathematical modelling—Fit three different *T*_piv_ and kept 29°C
Loggerhead (Zbinden et al., 2007)	Zakynthos	29.3	*T*_Piv_ previously calculated for same population (Mrosovsky et al., 2002)
Loggerhead (Öz et al., 2004)	Turkey	29	*T*_piv_ previously calculated for same population (Kaska et al., 1998)
Loggerhead (Hanson et al., 1998)	Florida	29	*T*_piv_ previously calculated for same population (Mrosovsky, 1988)
Loggerhead (Tanner et al., 2019)	Cabo Verde	29.25	*T*_piv_ previously calculated for different population (Marcovaldi et al., 1997)
Loggerhead (Jribi & Bradai, 2014)	Tunisia	29.7	*T*_piv_ previously calculated for different population (Mrosovsky et al., 2002)

The approach of using a pivotal temperature proxy during the middle third of incubation is now widely regarded to be too oversimplified, as the thermosensitive period is dependent on development rates, which are not linearly related to temperature, particularly under variable thermal environments (Georges et al., 2005; Massey et al., 2019). Importantly, it also does not account for individual variation in, or the adaptive potential of the pivotal temperature. Other measures that correlate with sex ratio, such as incubation duration, have also been used, but estimates tend to vary dependent on the proxy used (Fuentes et al., 2017). For instance, Fuentes et al. (2017) demonstrated that estimating the sex ratio of natural nests using the constant temperature equivalent (which converts natural fluctuating incubation temperatures into a constant value to be compared to temperatures produced under lab conditions (Georges et al., 2005)) during the middle third of incubation, and assuming a pivotal temperature of 29.12**°**C, predicted an average sex ratio of 5.99% (95% CI: <0.1%–37%) male. This estimate increased to 9.97% (95% CI: <0.1%–86.20%) male when using incubation duration as a proxy for the same nests. While the use of proxies has contributed to our general understanding of sex ratio distributions, their reliance on pre‐existing thermal response curves makes them unable to detect real‐time deviation from such curves that might originate from different elements of the adaptive potential of sea turtles—conceivably with major consequences to past and future management decisions.

## 8 TSD IN SEA TURTLES: ADAPTIVE POTENTIAL

To date, attention on the impact of global warming on the sex ratios of sea turtles has largely focused on how plastic behaviours can avoid extreme sex ratio biases. Such behaviours include nest site selection (Patrício et al., 2017; Reneker & Kamel, 2016), nest depth (Refsnider, Bodensteiner, et al., 2013; Refsnider, Jeanine, et al., 2013) and phenological shifts (Mazaris et al., 2013). For instance, with rising temperatures there is evidence that nesting seasons start earlier and can be more protracted for loggerhead turtles nesting in North Carolina and Greece (Hawkes, Broderick, Coyne, et al., 2007; Hawkes, Broderick, Godfrey, et al., 2007; Mazaris et al., 2013; Patel et al., 2016; Weishampel et al., 2004). From a global perspective, there is a significant negative relationship between the dates of first nesting for populations of loggerhead sea turtles across their nesting distribution, and the sea surface temperature at the beginning of the nesting season (Mazaris et al., 2013). Despite evidence of phenological variation, the sole extent of such shifts is unlikely to be sufficient to keep up with the rate of contemporary climate change (Monsinjon et al., 2019; Telemeco et al., 2013).

Consequently, mechanisms other than behavioural adjustments may prove vitally important for sea turtles to respond to climate change. Local adaptation, which, in the case of TSD, would involve populations adjusting thermal response curves to match the changing thermal environment of their nesting sites, could be a fundamental part of this process. Patterns of local adaptation have been shown to evolve from the philopatric nature of sea turtles, as previously demonstrated for immune genes (Stiebens et al., 2013) and even feeding strategies (Cameron et al., 2019). For instance, green sea turtle hatchlings from dark sand (high temperature) beaches on Ascension Island grow faster and have higher levels of hatching success than those from nearby white sand (cool) beaches when exposed to hot artificial incubation environments (Weber et al., 2012). Interestingly, no fine‐scale adaptation of the pivotal temperature was found between turtles nesting on these two beaches in a later study, showing the context dependence of the results (Tilley et al., 2019). On the other hand, flatback turtle *Natator depressus* eggs at tropical latitudes in Australia have high levels of tolerance to prolonged warming exposure up to 35**°**C during incubation, despite these temperatures often being lethal to other populations (Howard et al., 2015; Maulany et al., 2012). The pivotal temperatures of three genetically distinct flatback turtle populations also vary by 1.5°C, being highest in the population nesting under the warmest conditions (Bentley et al., 2020). These records of adaptation to local thermal environments provide evidence that the temperature sensitive triggers of the molecular pathways controlling sex determination can evolve (e.g. Ewert et al., 2005).

There is very little information on whether maternally derived sex steroid hormones can contribute to local adaptation, or whether they can introduce plasticity to the sea turtle TSD mechanism. This lack of attention to the evolvability of the endocrine system is unwarranted given the evidence from other TSD species (Bowden et al., 2000; Ewert et al., 2005). Experimental oestradiol treatment of olive ridley turtle eggs at male‐producing temperatures can feminize gonads (Merchant‐Larios et al., 1997), disrupt testis differentiation (Díaz‐Hernández et al., 2015), reduce cell proliferation (Díaz‐Hernández et al., 2017), delay *Sox9* inhibition and delay the upregulation of *FoxL2* and aromatase (Díaz‐Hernández et al., 2015). Furthermore, emerging research from a natural, in situ experiment that controlled for temperature found that the ratio of maternally derived oestradiol: testosterone in the yolk of loggerhead sea turtle eggs correlated with the sex ratio of nests independently of temperature (Lockley et al., 2020). Research that aims to evaluate the elements of the TSD mechanism that can evolve and locally adapt will be fundamental for revealing the adaptive potential of the TSD mechanism in sea turtles.

## 9 IMPLICATIONS FOR CONSERVATION MANAGEMENT

Global efforts dedicated to the conservation of sea turtles are extensive, with hundreds of grassroot, national and international projects working to stop the decline of populations and promote their persistence. Such work frequently focuses on the protection of nesting beaches (Fuentes et al., 2012; Hamann et al., 2010). Along with the benefits of accessibility, logistical advantages and low‐cost options, protection at this point in the life cycle can reduce threats from, for example, poaching of eggs and adult turtles (Senko et al., 2014; Tomillo et al., 2008), tidal inundation (Varela et al., 2019) and coastal development (Kaska et al., 2013; Von Holle et al., 2019).

A common conservation approach to mitigate the effects of human‐induced stressors such as coastal development and poaching is to relocate egg clutches into in situ hatcheries, where nests are protected and monitored (Mrosovsky, 2006, 2008; Pfaller et al., 2009; Pike, 2008; Tuttle & Rostal, 2010). However, these relocations should not be performed lightly, as shading, substrate properties and depth can be different from the nesting beach, and can all substantially alter the local thermal regimes (DeGregorio & Williard, 2011; Morreale et al., 1982; Tuttle & Rostal, 2010). The differences between temperatures in hatcheries and in situ sea turtle nests vary between locations. For instance, no difference in temperature was recorded during the thermosensitive period between relocated and in situ loggerhead sea turtle nests in North Carolina, but over the entire incubation period nests in the hatchery were exposed to higher overall temperatures, and hatchlings from these nests emerged sooner (DeGregorio & Williard, 2011). However, this was not observed in Georgia, where there were no significant differences in temperatures, size or incubation duration between relocated and in situ nests, but there was reduced survival in the nests that had been relocated (Tuttle & Rostal, 2010). If nest relocation is undertaken, then continuous evaluation of incubation temperatures, durations and the effects on hatchling development and fitness should always be conducted.

Crucially, if done incorrectly, relocation may interfere with natural selection for traits that will help sea turtles adjust to new temperature regimes, or reduce the effectiveness of natural buffering mechanisms, as climate change progresses (Mrosovsky, 2006; Pfaller et al., 2009). This effect may be particularly strong for these highly philopatric species, as they are the ones predicted to have evolved the strongest signature of local adaptation (Baltazar‐Soares et al., 2020; Stiebens et al., 2013). Specifically, human manipulation of nest temperatures might dampen the effects of natural selection on developing embryos, by reducing selection on the genomic mechanisms that confer thermal tolerance and a higher pivotal temperature. Alternatively, if plastic phenotype–environment matching were occurring, either in the form of maternal hormone transfer or nesting behaviour, intentionally modifying incubation conditions would cause a human‐induced thermal mismatch between the clutch and its optimal conditions.

It is thus important that if nest relocation is necessary to protect from stressors such as inundation or predation, this is done in a manner that matches the thermal environment that embryos would have experienced had eggs remained in situ. This includes taking all possible care to relocate nests to similar substrates in the vicinity of the nesting beach, in a nest cavity of the same depth and shape as originally dug by the female, with similar hydric properties. By doing this, conservationists will ensure that any behaviour is accounted for, while ensuring that unseen physiological plasticity such as hormone transfer will act in the direction of natural selection. In addition, in light of our current lack of knowledge on accurate sex ratios and adaptive potential, relocating nests with the direct intention of manipulating temperature regimes (in an attempt to mitigate against global warming itself) risks preventing populations from adapting to conditions naturally. This leaves conservationists in a position where leaving egg clutches exposed to increasing temperatures is not viable, but altering incubation conditions by reducing temperatures may also have long‐term negative consequences, whereby local adaptation is prevented or reduced. For scientists interested in TSD, focusing on developing nonlethal sexing methods that can be applied at a large scale would be extremely useful to guide such future conservation decisions.

## 10 CONCLUSIONS

Sea turtle conservation managers and scientists face difficult management choices as global warming progresses, and tools are still missing to make fully informed decisions about mitigation strategies. Barriers to determining neonate sex have limited our understanding of primary sex ratios in this taxon, and this knowledge gap must rapidly be overcome. It is likely that sea turtles have evolved heritable mechanisms, whether behavioural, genetic, epigenetic or physiological, to respond to climate change, but these are not currently quantified.

To inform future decisions, we must accept it is not sufficient to estimate sex ratios based on theoretical pivotal temperatures from distant populations. We must begin to monitor sex ratios in a high throughput manner to quantify the adaptive potential of these species. This will enable us to assess the thermal response curves of populations and nesting aggregations, along with their variation across time and space. How this variation is maintained is an important question in the light of climate change and the underlying mechanisms will need to be clarified.

## CONFLICT OF INTEREST

The authors declare no conflicts of interest.

## Data Availability

Data sharing not applicable to this article as no datasets were generated or analysed during the current study.
